# Genetic Diversity and Selection of *Plasmodium vivax* Apical Membrane Antigen-1 in China–Myanmar Border of Yunnan Province, China, 2009–2016

**DOI:** 10.3389/fcimb.2021.742189

**Published:** 2022-01-05

**Authors:** Yan-Bing Cui, Hai-Mo Shen, Shen-Bo Chen, Kokouvi Kassegne, Tian-Qi Shi, Bin Xu, Jun-Hu Chen, Jia-Hong Wu, Yue Wang

**Affiliations:** ^1^ National Institute of Parasitic Diseases, Chinese Center for Diseases Control and Prevention (Chinese Center for Tropical Diseases Research), Shanghai, China; ^2^ National Health Commission of the People’s Republic of China (NHC) Key Laboratory of Parasite and Vector Biology, Shanghai, China; ^3^ World Health Organization (WHO) Collaborating Center for Tropical Diseases, Shanghai, China; ^4^ National Center for International Research on Tropical Diseases, Shanghai, China; ^5^ School of Global Health, Chinese Center for Tropical Diseases Research, Shanghai Jiao Tong University School of Medicine, Shanghai, China; ^6^ Key Laboratory of Environmental Pollution Monitoring and Disease Control, Ministry of Education, Guizhou Medical University, Guiyang, China; ^7^ Department of Parasitology, Provincial Key Laboratory of Modern Pathogen Biology, Guizhou Medical University, Guiyang, China; ^8^ Institute of Parasitic Diseases, School of Basic Medical Sciences and Forensic Medicine, Hangzhou Medical College, Hangzhou, China

**Keywords:** *Plasmodium vivax*, apical membrane antigen-1, genetic diversity, positive selection, vaccine, China–Myanmar border area

## Abstract

*Plasmodium vivax* apical membrane antigen-1 (PvAMA-1) is an important vaccine candidate for vivax malaria. However, antigenic variation within PvAMA-1 is a major obstacle to the design of a global protective malaria vaccine. In this study, we analyzed the genetic polymorphism and selection of the PvAMA-1 gene from 152 *P. vivax* isolates from imported cases to China, collected in the China–Myanmar border (CMB) area in Yunnan Province (YP) during 2009–2011 (*n* = 71) and 2014–2016 (*n* = 81), in comparison with PvAMA-1 gene information from Myanmar (*n* = 73), collected from public data. The overall nucleotide diversity of the PvAMA-1 gene from the 152 YP isolates was 0.007 with 76 haplotypes identified (*Hd* = 0.958). Results from the population structure suggested three groups among the YP and Myanmar isolates with optimized clusters value of *K* = 7. In addition, YP (2014–2016) isolates generally lacked some *K* components that were commonly found in YP (2009–2011) and Myanmar. Meanwhile, PvAMA-1 domain I is found to be the dominant target of positive diversifying selection and most mutation loci were found in this domain. The mutation frequencies of D107N/A, R112K/T, K120R, E145A, E277K, and R438H in PvAMA-1 were more than 70% in the YP isolates. In conclusion, high genetic diversity and positive selection were found in the PvAMA-1 gene from YP isolates, which are significant findings for the design and development of PvAMA-1-based malaria vaccine.

## Introduction

Malaria is still a serious infectious disease that threatens human health and affects social and economic development in the world. According to the World Health Organization (WHO), by 2019, there were 229 million malaria cases, an increase of one million over 2018 and 409,000 malaria deaths worldwide (WHO, 2020). *Plasmodium vivax* is one of the five species of *Plasmodium* that regularly infect humans and cause malaria, and is the most widely distributed human malaria species outside the African continent with an estimated 2.5 billion people at risk of infection (Vogel, 2013; Howes et al., 2016). *Plasmodium vivax* is widely prevalent in parts of Asia (Flannery et al., 2019), especially, in some countries bordering China, such as Myanmar, Laos, and Vietnam (von Seidlein et al., 2019; Brashear et al., 2020).

*Plasmodium vivax* was once a serious epidemic in China (Feng et al., 2015). Although there were no local cases of *P. vivax* in China since 2017 (Lai et al., 2019), there is still a huge risk of re-emerging cases due to the existence of *Anopheles* vectors (Zhang et al., 2018). In the China–Myanmar border (CMB) area, with the development of the Belt and Road Initiative, the risk of imported malaria cases to China is increasing (Lai et al., 2019). Therefore, studies to dissect the genetic backgrounds of *P. vivax* in the CMB area are of great significance to provide information for the control and elimination of vivax malaria in Myanmar and other related countries.

In recent years, drug resistance to *P. vivax* was frequently reported (Imwong et al., 2007; Lu et al., 2010; Dayananda et al., 2018). Meanwhile, there are many other factors which contribute to the difficulties of *P. vivax* control, such as hypnozoites (White and Imwong, 2012), early gametocytogenesis, frequent low parasitemias, high infectivity to mosquitoes, and shorter development cycle in the vector host compared with other species of *Plasmodium* (Mueller et al., 2009; Mueller and Adams, 2017). Thus, the development of a stable and effective vaccine has been proposed as a possible aid to drugs for effective control and elimination of vivax malaria. *Plasmodium vivax* apical membrane antigen-1 (PvAMA-1) is an important candidate for malaria vaccine (Mueller et al., 2015; Beeson et al., 2019). Apical membrane antigen-1 (AMA-1) is expressed in the microneme of apicomplexan parasites and is present in all *Plasmodium* species (Bittencourt et al., 2020). It is a type I transmembrane protein binding with rhoptry neck proteins (Srinivasan et al., 2011). AMA-1 is involved in merozoite reorientation and tight junction formation during the invasion process (Gaur et al., 2004; Richard et al., 2010; Lamarque et al., 2011) and is essential for parasite survival (Triglia et al., 2000). It has been reported that antibodies against the ectodomain of *Plasmodium falciparum* AMA-1 (PfAMA-1) can inhibit erythrocyte invasion, and its immunization protects against malaria infection (Remarque et al., 2008; Kusi et al., 2009). The extracellular domain of AMA-1 is divided into three subdomains referred to as domain I, domain II, and domain III based on the conserved cysteine residues (Pizarro et al., 2005). Domain I and domain II cover the polymorphic regions and have been shown to be the major targets that elicit inhibitory responses (Remarque et al., 2008). In general, the formation of moving junction between merozoite and erythrocyte is the key procedure for the successful invasion of host cells by the parasite, and the mechanism includes the interaction between the conserved hydrophobic groove in domain II loop of AMA-1 and the conserved rhoptry neck protein 2 (RON2) loop (Bargieri et al., 2013). PvAMA-1, therefore, becomes an important immune target (Múfalo et al., 2008). Meanwhile, due to the highly polymorphic feature of the PvAMA-1 gene, it has been used as molecular marker for population genetic studies (Joshi, 2003). However, antigenic variation is a major challenge in the design of protective malaria vaccine (Esmaeili Rastaghi et al., 2014). Recently, a report on genetic diversity of *Pvama-1* in India also showed this trend (Kale et al., 2021).

In the present study, the genetic diversity and selection of PvAMA-1 gene in the CMB area were investigated in *P. vivax* isolates collected from Yunnan Province (YP) in China during 2009–2011 and 2014–2016 and compared with the public PvAMA-1 gene information from Myanmar, to advance our knowledge of the rational design of vivax malaria vaccine.

## Materials and Methods

### Ethics Statement

This study was conducted according to the principles expressed in the Declaration of Helsinki. Before blood collection, the study protocol, potential risks, and benefits were explained to the participants, and written informed consent was obtained from all adult participants and from the parents or legal guardians of children. Blood was collected following institutional ethical guidelines reviewed and approved by the Ethics Committee at the National Institute of Parasitic Diseases, Chinese Center for Disease Control and Prevention (no. 20120826).

### Sample Collection and DNA Extraction

**Figure 1:**
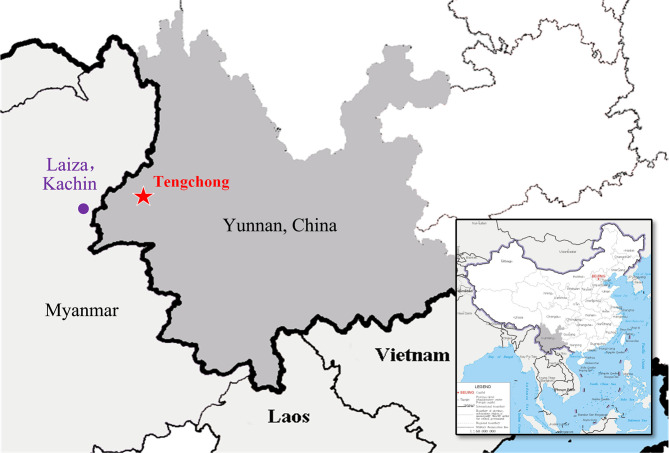
Geographic map of *Plasmodium vivax* samples collection. The area where samples were collected (Tengchong County, Yunnan Province, China) for this study is indicated in a red pentagram (China map version GS(2019)1652 downloaded from URL: http://bzdt.ch.mnr.gov.cn/).

A total of 180 blood samples of malaria patients infected with *P. vivax* were collected from the CMB area in YP (China) during 2009–2011 (*n* = 77) and 2014–2016 (*n* = 103) (**Figure 1**). All samples were stained by Giemsa and verified by microscopic examination then confirmed for single infection with *P. vivax* by nested PCR (Zhou et al., 2014). Genomic DNA was extracted from whole blood using the DNeasy Blood & Tissue Kit (Qiagen, Germany) as previously reported (Chen et al., 2017; Kassegne et al., 2020).

### PCR Amplification and Sequencing

To identify *Plasmodium* species, nested PCR was performed as previously described (Zhou et al., 2014). In the first PCR, the DNA fragment was amplified by 2×Taq PCR MasterMix (Tiangen Biotech, Beijing, China) using the primers rPLU1 5′-TCAAAGATTAAGCCATGCAAGTGA-3′ and rPLU5 5′-CCTGTTGTTGCCTTAAACTTC-3′. One microliter of template DNA was added to a 20-μl PCR mixture consisting of 0.4 μM of each primer, 10 μl 2×Taq PCR MasterMix (Tiangen Biotech, Beijing, China) containing 0.1 U Taq polymerase/μl, 500 μM deoxynucleotide triphosphates (dNTP), 3 mM MgCl_2_, 100 mM KCl, and 20 mM Tris–HCl, pH 8.3. The cycling parameters to amplify the fragments were as follows: initial denaturation at 94°C for 5 min; 30 cycles of 94°C for 30 s, 55°C for 30 s, and 72°C for 1 min; followed by a final extension at 72°C for 5 min. One microliter of the first PCR product was used in the second amplification. Conditions and concentrations used for the second amplification were identical to those used for the first, except that rVIV1/rVIV2 (rVIV1 5′-CGCTTCTAGCTTAATCCACATAACTGATAC-3′, rVIV2 5′-ACTTCCAAGCCGAAGCAAAGAAAGTCCTTA-3′) were used as primers and amplification was performed over 35 cycles. The size of the DNA target amplified by these outer primers is about 1,600–1,700 bp and that by the inner primers is 121 bp. PCR products were qualitatively analyzed on 2% agarose gel and were sent to Beijing Genomics Institution (BGI, Shenzhen, China) for sequencing.

The PvAMA-1 fragment was amplified by PrimerSTAR Max DNA Polymerase (Takara, Japan) using the primers SeqF1 5′-CCCTACCAGCGGCTACTTC-3′ and SeqR1 5′-CGTTTGCTTGGCCAACTC-3′. All PCR amplifications were performed in a 50-μl PCR reaction volume containing 0.4 μM of each primer pair, 1.5 mM MgCl_2_, 1× PCR buffer (50 mM KCl, 10 mM Tris–Cl, pH 8.3), 0.2 mM dNTPs, and 0.5 unit of DNA polymerase. The cycling parameters to amplify the fragments were as follows: initial denaturation at 98°C for 5 min, 35 cycles of denaturation at 98°C for 10 s, annealing at 69°C for 15 s, extension at 68°C for 1 min 30 s, and a final extension at 68°C for 5 min. The PCR fragments were about 1,900 bp in size and contained all the gene fragments of PvAMA-1 (1,689 bp). The PCR products were qualitatively analyzed on 1% agarose gel and were sent to Beijing Genomics Institution (BGI, Shenzhen, China) for sequencing. All unique mutations were carefully checked, and ambiguous bases were confirmed by resequencing. Meanwhile, a set of 73 sequences of *Pvama-1* (1,290 bp) isolates from Myanmar was downloaded from NCBI (KX495505–KX495577) (Zhu et al., 2016).

### Data Analysis

All sequences were assembled and aligned by DNAMAN. After all the sequences were aligned, data were trimmed to 1,689 bp, which contains the full coding sequence of PvAMA-1. We performed a multiple sequence alignment of the PvAMA-1 gene sequences using MEGA6 (Tamura et al., 2013). PvAMA-1 gene sequence PVP01_0934200.1 (1,689 bp) obtained from PlasmoDB (http://PlasmoDB.org) was used as reference.

After all the sequences were aligned, three domains of PvAMA-1 were also divided. These included domain I (462 bp, nucleotides 280–741), domain II (297 bp, nucleotides 793–1,089), and domain III (192 bp, nucleotides 1,162–1,353). To investigate the genetic polymorphism and selection of PvAMA-1 in different time periods in the CMB area, we used DnaSP (Librado and Rozas, 2009) to calculate the number of haplotypes (*H*), the mean value of nucleotide differences (*k*), nucleotide diversity (*π*), and haplotype diversity (Hd) as previously described (Murhandarwati et al., 2020), with a sliding window of 100 bp and step size of 25 bp for nucleotide diversity (*π*). KaKs_Calculator 2.0 software (Wang et al., 2010) was used to calculate the non-synonymous (Ka) and synonymous (Ks) substitution rates. The Ka and Ks values and Ka/Ks ratios were calculated based on a model-averaged method (Wang et al., 2010). Ka/Ks calculation was used to estimate the selection pressure of PvAMA-1 gene pairs. The algorithm was NG (Nei and Gojobori, 1986) and YN, which is an alternative model for NG (Yang and Nielsen, 2000). Additionally, Tajima’s *D* test was performed in DnaSP to evaluate the neutrality theory of evolution (with Fu and Li’s test as a double check). The probability of recombination between adjacent nucleotides per generation was calculated using DnaSP. The linkage disequilibrium (LD) between different polymorphic sites was computed based on the *D* and *R* ^2^ indices. The calculations performed through DnaSP were based on the default parameters.

The recombination region was calculated by Recombination Detection Program v.4.101 (Martin et al., 2015) with the MaxChi method (Maynard Smith, 1992). After removing the recombination block, a haplotype network based on the *P*v*ama-1* sequence was constructed using the NETWORK software Version 10200 with the median-joining method (Bandelt et al., 1999). To assess allele ancestry, STRUCTURE software was used to assess clustering of isolates under the ancestry model “Use Population Information to test for migrants” (Evanno et al., 2005). Six iterations for the numbers of clusters (*K*) from three to eight were run, each with a burning period of 5,000 steps and 10,000 Markov chain Monte Carlo iterations. The best *K* was selected as previously described (Evanno et al., 2005). The sequences of nine additional *P*v*ama-1* populations (Myanmar, KX495505–KX495577; Thailand, FJ784891–FJ785121; Korea, KM230319–KM230384; Sri Lanka, EF218679–EF218701; Iran, JX624732–JX624760; Papua New Guinea (PNG), KC702402–KC702503; India, MH657021–MH657120; Venezuela, EU346015–EU346087; and Brazil, MH049550–MH049589) were analyzed together (Gunasekera et al., 2007; Ord et al., 2008; Putaporntip et al., 2009; Arnott et al., 2013; Zakeri et al., 2013; Kang et al., 2015; Zhu et al., 2016; Bittencourt et al., 2020; Kale et al., 2021). Here, the India population (Kale et al., 2021) should be considered as a long-distance geography distribution outgroup since Kale et al. had exhibited the genetic difference between Myanmar and South Asia. Before the Network and Structure analyses, all the sequences of PvAMA-1 were analyzed and cut to 1,290 bp according to the fragment of PvAMA-1 isolates from Myanmar (Zhu et al., 2016) through MEGA6 and using Arlequin3.5 to analyze the molecular variance (AMOVA) to evaluate fixation (*F*_ST_) (Excoffier and Lischer, 2010). Meanwhile, the migration rate between YP and Myanmar populations was estimated by Migrate-n version 4.4.3 (Beerli, 2006).

## Results

Among the 180 blood samples infected with *P. vivax* from the CMB area during 2009–2011 (*n* = 77) and 2014–2016 (*n* = 103), 152 (71 and 81, respectively) samples were successfully sequenced for the PvAMA-1. The 152 patients, composed of 109 males and 43 females, whose blood samples successfully amplified the *Pvama-1*, aged from 6 to 66 years.

### Genetic Diversity of *Pvama-1* in *Plasmodium vivax* Isolates From the CMB Area

**Figure 2:**
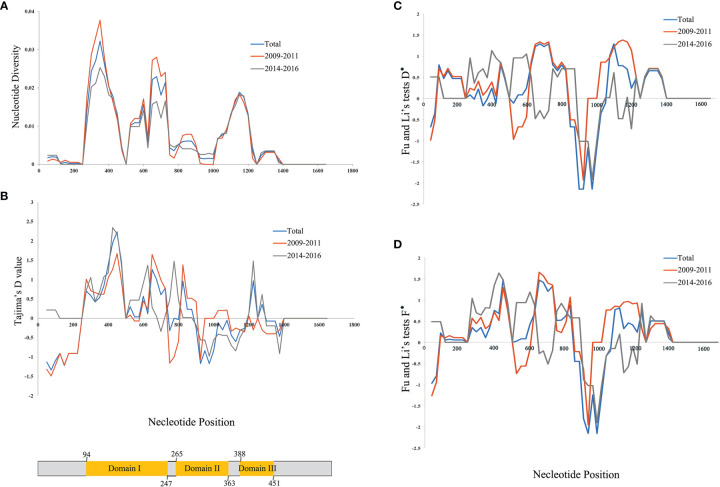
Nucleotide diversity for *Plasmodium vivax* apical membrane antigen-1 (PvAMA-1) in isolates from Yunnan Province (YP). **(A)** Position of PvAMA-1 nucleotide diversities. **(B)** Tajima’s *D* value for PvAMA-1. **(C)** D* value of Fu and Li’s tests for PvAMA-1. **(D)** F* value of Fu and Li’s tests for PvAMA-1. Blue, orange, and gray lines represent the different time periods, namely, Total, 2009–2011, and 2014–2016, of YP isolates, respectively. A scheme of the domains of PvAMA-1 is also shown (in yellow color) with amino acid positions indicated.

Of the 152 *Pvama-1* sequences from the CMB area, there were 76 haplotypes, giving an overall haplotype diversity (Hd) of 0.958 (**Table 1**). The average number of pairwise nucleotide differences (*k*) for the entire 1,689 bp in different time periods including the full sampling period (referred to as Total), 2009–2011, and 2014–2016 were 12.191, 12.933, and 10.814, respectively (**Table 1**). The number of haplotypes was 76, 54, and 23 for Total, 2009–2011, and 2014–2016, respectively (**Table 1**). A total of 61 single nucleotide polymorphisms (SNPs) were detected, including 54 SNPs in 2009–2011 and 47 SNPs in 2014–2016 (**Table 1**). Nucleotide diversity (*π*) of the YP samples from 2009 to 2011 and from 2014 to 2016 was 0.00766 and 0.00640, respectively, and that of the total samples was 0.00722 (**Table 1**). Nucleotide diversity (*π*) of the YP samples in three domains was 0.01701, 0.00482, and 0.00355, respectively, in which the *Pvama-1* domain I showed the highest genetic variation. Recently, Kale et al. (2021) reported the genetic diversity of PvAMA-1 in India and confirmed that the high genetic variation was observed in *Pvama-1* domain I. *π* values for 2009–2011 and 2014–2016 were ranging from 0.000 to 0.03771 (350–380 bp) and from 0.000 to 0.02533 (350–380 bp), respectively (**Figure 2A**).

**Table 1:** Nucleotide diversity and summary statistics of PvAMA-1 in 152 *Plasmodium vivax* isolates from the China–Myanmar border area between different time periods.

Samples	*n*	*k*	*H*	Hd ± SD	*S*	Sv	Sp	*η*	*π*	Ka	Ks	Ka/Ks	*D* ^a^	D*^b^	F*^c^
Total	152	12.191	76	0.958 ± 0.009	61	9	52	64	0.00722	0.00959	0.005056	1.89662*	0.205	0.522	0.461
Domain I		7.861	53	0.948 ± 0.010	32	4	28	33	0.01701	0.019427	0.019638	0.98924	0.979	0.666	0.955
Domain II		1.432	17	0.789 ± 0.027	9	3	6	9	0.00482	0.005814	0.005051	1.15114	−0.257	−1.102	−0.957
Domain III		0.682	4	0.531 ± 0.038	3	0	3	3	0.00355	0.005539	0	NA	0.456	0.793	0.807
2009–2011	71	12.933	54	0.990 ± 0.004	54	7	47	57	0.00766	8.04741	2.02589	3.97228*	0.321	0.880	0.794
Domain I		8.943	42	0.982 ± 0.005	32	5	27	33	0.01936	7.07866	1.51383	4.67598*	0.991	0.537	0.846
Domain II		1.475	13	0.836 ± 0.026	6	1	5	6	0.00497	6.77261	2.5713	2.63392*	0.448	0.236	0.359
Domain III		0.471	3	0.299 ± 0.067	3	0	3	3	0.00245	6.40604	0.741938	8.63419*	-0.463	0.857	0.525
2014–2016	81	10.814	23	0.859 ± 0.026	47	6	41	49	0.00640	8.07485	2.07535	3.89083*	0.311	0.421	0.452
Domain I		6.478	19	0.845 ± 0.027	24	2	22	25	0.01402	7.17737	1.5293	4.69323*	0.877	0.754	0.956
Domain II		1.317	11	0.710 ± 0.047	8	2	6	8	0.00444	6.7826	1.41508	4.79308*	−0.457	−0.288	−0.408
Domain III		0.804	4	0.639 ± 0.030	3	0	3	3	0.00419	6.50997	0.777672	8.3711*	0.618	0.845	0.906

The total sequenced region includes codons 1 to 479: domain I codons 94 to 247 (nt 280–741), domain II codons 265 to 363 (nt 793–1,089), and domain III codons 388 to 451 (nt 1,162–1,353).

n, number of samples; k, the average number of nucleotide differences; H, number of haplotypes; Hd, haplotype diversity; SD, standard deviation; S, number of polymorphic (segregating) sites; Sv, the number of singleton sites; Sp, the number of informative-parsimonious sites; η, the total number of mutations; π, nucleotide diversity; Ka, the rates of non-synonymous substitutions; Ks, the rates of synonymous substitutions; Ka/Ks, the ratio of non-synonymous to synonymous mutations; D, Tajima’s D test; D*, Fu and Li’s D* value; F*, Fu and Li’s F* value; NA, cannot be calculated.

*P < 0.05. ^a,b,c^P > 0.10.

In order to assess the neutral evolving of PvAMA-1 and its three domains, Tajima’s *D* test was performed. Tajima’s *D* value of the full PvAMA-1 fragment was 0.205 for Total, 0.321 for 2009–2011, and 0.311 for 2014–2016 (*P* > 0.1; **Table 1**). However, the Tajima’s *D* values obtained for the three domains were obviously different (**Table 1**). The Tajima’s *D* value of domain I was 0.979 for Total, 0.991 for 2009–2011, and 0.877 for 2014–2016. The Tajima’s *D* value of domain II was −0.257, 0.448, and −0.457 for Total, 2009–2011, and 2014–2016, respectively. The Tajima’s *D* value of domain III was 0.456 for Total, −0.463 for 2009–2011, and 0.618 for 2014–2016 (*P* > 0.1; **Table 1**). These indicate that Tajima’s *D* test showed opposite selection directions for different domains of PvAMA-1 across different times (**Figure 2B**). Domain II (from nucleotide positions 795 to 1,089) showed positive Tajima’s *D* value for 2009–2011 and negative value for 2014–2016, which are consistent with the results in Fu and Li’s tests (**Table 1** and **Figures 2C, D**). For the domain III fragment, Tajima’s *D* value was negative for 2009–2011 and positive for 2014–2016, which were in partial deviation from the analysis results of Fu and Li’s tests with values all positive (**Table 1** and **Figures 2C, D**). Meanwhile, a sliding window plot depicted significant positive values (nt 401–500, Tajima’s *D*: 2.233, *P* < 0.05) in domain I of YP samples, suggesting positive diversifying selection in this region.

**Figure 3:**
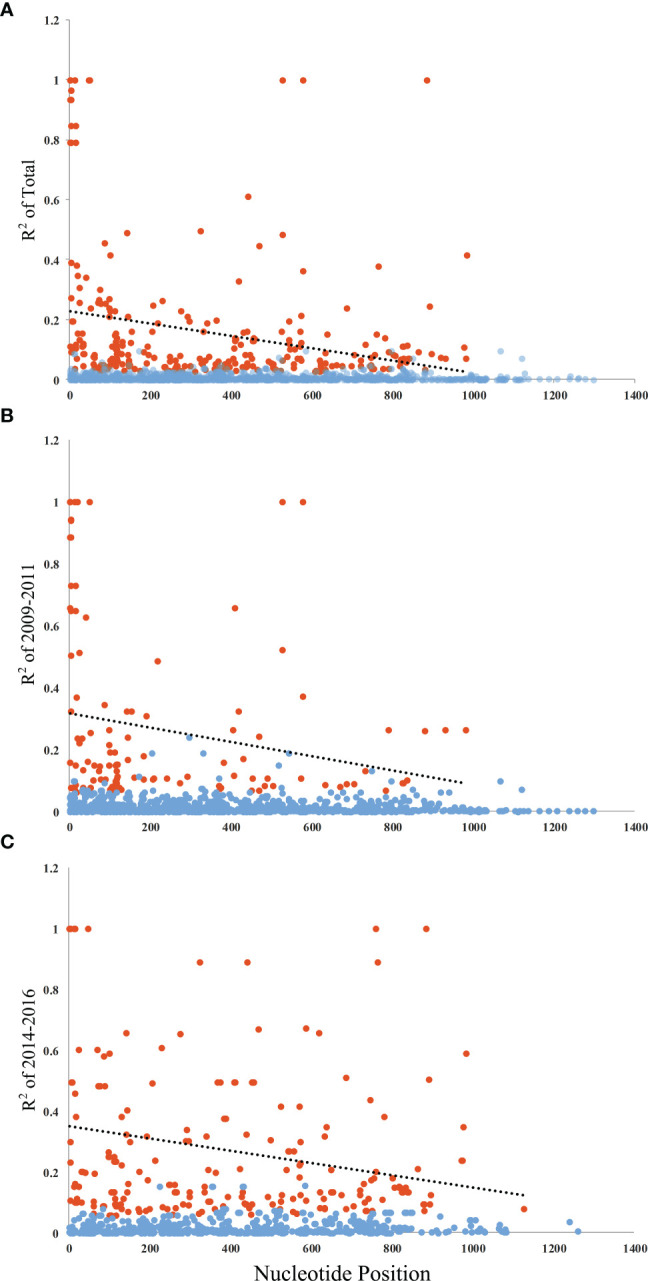
Linkage disequilibrium (LD) of PvAMA-1 in isolates from YP. LD across the PvAMA-1 gene in the isolates was calculated using *R* ^2^. **(A)** *R* ^2^ for PvAMA-1 gene of Total isolates. **(B)** *R* ^2^ for PvAMA-1 gene of 2009–2011 isolates. **(C)** *R* ^2^ for PvAMA-1 gene of 2014–2016 isolates. Significant LD values among samples are shown as calculated by Fisher’s exact test. Trace line represents the regression line. Orange and blue dots represent significant and non-significant *R* ^2^ values, respectively.

**Figure 4:**
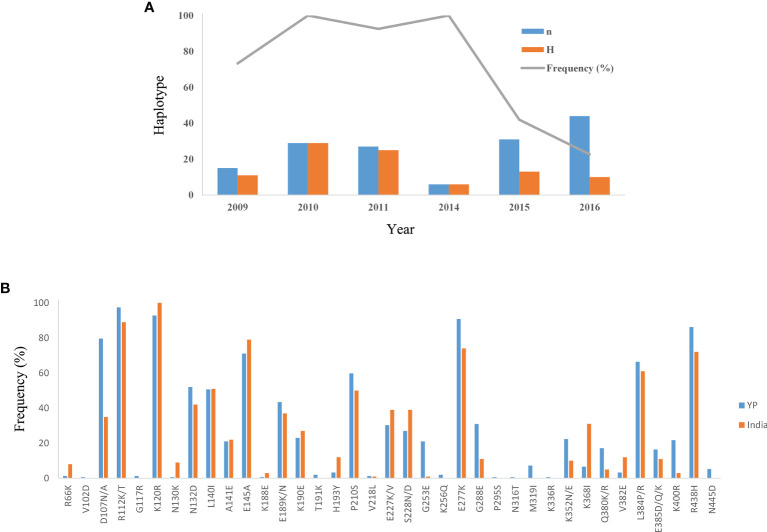
Variation of haplotype frequency of YP isolates and distribution of amino acid mutation sites of YP samples in the Indian population. **(A)** The change in the frequency of haplotypes over time in YP isolates. *n*, number of samples; *H*, number of haplotypes; Frequency (%), frequency of haplotypes. **(B)** The distribution of amino acid mutation sites (R66–N445) of YP samples in the Indian population.

The Ka/Ks in PvAMA-1 for the Total was 1.89662 (YN, *P* < 0.05). The Ka/Ks values were 3.97228 for 2009–2011 and 3.89083 for 2014–2016 (YN, *P* < 0.05), suggesting a significant positive selection for PvAMA-1 of *P. vivax* populations in the CMB area during these times (**Table 1**). The LD index (*R* ^2^) also declined with distance, suggesting that intragenic recombination may also contribute to the PvAMA-1 diversity (**Figure 3**). Furthermore, we performed the LD test on different time periods and found the 2009–2011 subpopulation under similar pattern for LD SNP pairs with the whole population (**Figure 3**). In contrast, the 2014–2016 subpopulation showed more LD SNP pairs than the 2009–2011 subpopulation did, which may suggest less recombination ratio from the founder effect (**Figure 3**). Meanwhile, we analyzed the change in the frequency of haplotypes over time, and the result showed that the frequency of haplotypes decreased significantly in 2015 and 2016 samples (**Figure 4A**).

### Mutations of PvAMA-1 in Isolates From the CMB

A total of 40 amino acid mutation sites were found in YP samples. Among them, 37 and 29 amino acid mutation sites were found in 2009–2011 and 2014–2016 samples, respectively. In the Total samples, the mutation frequencies of 19 mutation sites were less than 10%; for 11 mutation sites, 15%–45%; and for 10 mutation sites, more than 50% (**Table 2**). More importantly, the mutation frequencies of D107N/A, R112K/T, K120R, E145A, E277K, and R438H were more than 70% (**Table 2**). Some mutation sites with low frequency in the 2009–2011 samples disappeared in the 2014–2016 samples (**Table 2**, **Table S1**). There were also three new mutation sites in the 2014–2016 samples, in which the mutation frequencies of N316T, M319I, and K336R were 0.66%, 7.24%, and 0.66%, respectively (**Table 2**). Furthermore, there were 19 mutation sites in domain I of PvAMA-1, in which the most mutated amino acid sites were distributed. The number of amino acid mutation sites in domains II and III were seven and three, respectively.

**Table 2:** Amino acid variations of PvAMA-1 in the CMB area.

Codons	ns	Frequency (%)	Position
		YP (*n* = 152)	
A12G	1	0.66	
Q25H/K	2/11	8.55	
G42V	2	1.32	
R66K	2	1.32	
V102D	1	0.66	Domain I
D107N/A	5/116	79.61	Domain I
R112K/T	92/56	97.37	Domain I
G117R	2	1.32	Domain I
K120R	141	92.76	Domain I
N130K	1	0.66	Domain I
N132D	79	51.97	Domain I
L140I	77	50.66	Domain I
A141E	32	21.05	Domain I
E145A	108	71.05	Domain I
K188E	1	0.66	Domain I
E189K/N	56/10	43.42	Domain I
K190E	35	23.03	Domain I
T191K	3	1.97	Domain I
H193Y	5	3.29	Domain I
P210S	91	59.87	Domain I
V218L	2	1.32	Domain I
E227K/V	6/40	30.26	Domain I
S228N/D	2/39	26.97	Domain I
G253E	30	19.74	
K256Q	3	1.97	
E277K	138	90.79	Domain II
G288E	47	30.92	Domain II
P295S	1	0.66	Domain II
N316T	1	0.66	Domain II
M319I	11	7.24	Domain II
K336R	1	0.66	Domain II
K352N/E	24/10	22.37	Domain II
K368I	10	6.58	
Q380K/R	7/19	17.11	
V382E	5	3.29	
L384P/R	41/60	66.45	
E385D/Q/K	13/10/2	16.45	
K400R	33	21.71	Domain III
R438H	131	86.18	Domain III
N445D	8	5.26	Domain III

ns, number of mutant isolates; n, number of isolates.

Here, we also analyzed the distribution of these mutation sites of YP samples in other groups (**Table S1**). In the 37 mutation sites of YP samples, the lowest coincidence rate was found in the Korean samples and the highest coincidence rate in the Iran and India samples (**Table S1**). At the same time, we also calculated the mutation ratio of these mutation sites of YP samples in the Indian population (**Figure 4B**). It showed that the relatively preserved amino acid changes found in the YP PvAMA-1 were well-conserved in India (**Figure 4B**).

### Genetic Differentiation, Haplotype Network, and Structure Analyses of PvAMA-1

A total of 889 *Pvama-1* sequences, containing YP, Myanmar, Thailand, South Korea, Papua New Guinea, Sri Lanka, Iran, India, Venezuela, and Brazil populations, were analyzed and cut to 1,290 bp through MEGA6. The level of genetic differentiation of *Pvama-1* was estimated by *F*_ST_ values. In general, the *F*_ST_ values of <0.05, 0.05–0.15, 0.15–0.25, and >0.25 indicate little, moderate, great, and very great genetic differentiation, respectively (Balloux and Lugon-Moulin, 2002). The YP, Sri Lanka, and Brazil isolates showed great differentiation with *F*_ST_ values of 0.15269 and 0.24836, respectively. The very great differentiation was found between YP isolates and Korea isolates (the value of *F*_ST_ is 0.3761) (**Table 3**). The differentiation between YP isolates and other isolates was moderate (**Table 3**).

**Table 3:** Estimation of genetic differentiation (*F*_ST_) of the *Pvama-1* among geographical populations.

Population	YP	Sri Lanka	Venezuela	Thailand	Iran	PNG	Korea	Myanmar	Brazil	India
YP (*n* = 152)	0.00000									
Sri Lanka (*n* = 23)	0.15269	0.00000								
Venezuela (*n* = 73)	0.12335	0.2536	0.00000							
Thailand (*n* = 231)	0.06022	0.1947	0.15645	0.00000						
Iran (*n* = 29)	0.05518	0.09445	0.09774	0.12106	0.00000					
PNG (*n* = 102)	0.07025	0.21904	0.23074	0.16569	0.10904	0.00000				
Korea (*n* = 66)	0.3761	0.5603	0.47258	0.43773	0.39093	0.40805	0.00000			
Myanmar (*n* = 73)	0.06658	0.23478	0.12237	0.03273	0.1143	0.18777	0.46368	0.00000		
Brazil (*n* = 40)	0.24836	0.41572	0.25526	0.2833	0.16918	0.2868	0.4053	0.2788	0.00000	
India (*n* = 100)	0.06658	0.05684	0.1311	0.13641	−0.00506	0.10625	0.38537	0.13389	0.23274	0.00000

n, number of samples.

P < 0.05.

**Figure 5:**
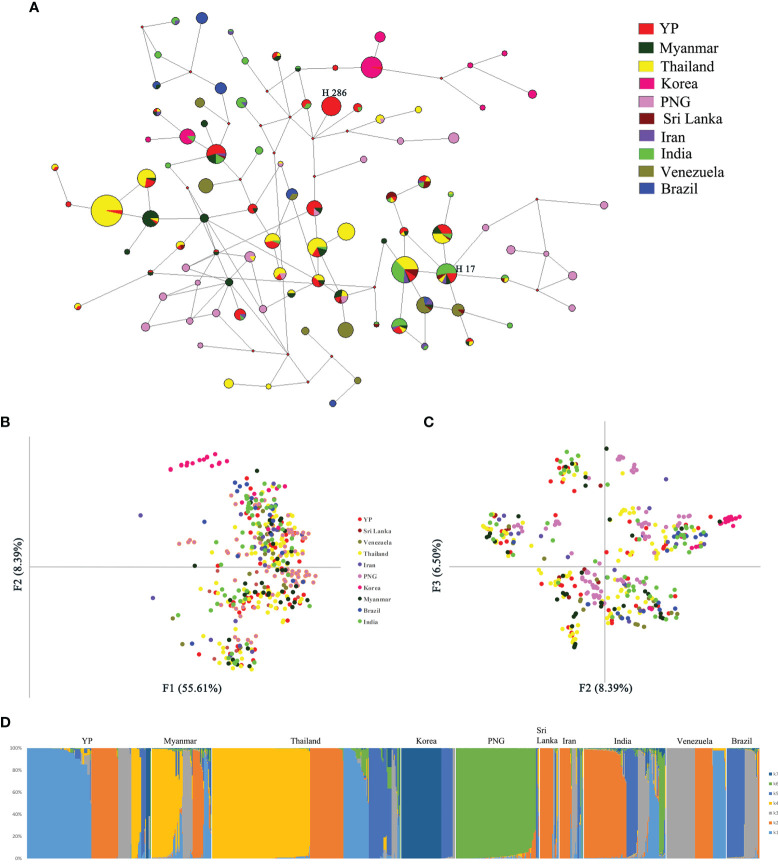
Network, principal component, and structure analyses of PvAMA-1 in global isolates. **(A)** The proportion of Pv*ama-1* haplotype variations observed in different populations. Samples are colored according to different populations. **(B)** Principal component analysis with F1 and F2. **(C)** Principal component analysis with F2 and F3. **(D)** Structure analysis of the full set of variation loci from all isolates. Cluster for each isolate was assessed according to an optimized cluster value of *K* = 7.

One recombination event of YP samples was detected by Recombination Detection Program v.4.101, containing a 602-bp fragment, located in 493–1,094 bp of *Pvama-1*. After removing the recombination block, a total of 889 sequences from the global populations, length 688 bp, were analyzed by the NETWORK software. Network analysis results for PvAMA-1 populations showed that there was an obvious cluster of populations of global samples, except those from Korea and PNG which were partially separated (**Figure 5A**). Among them, the haplotype sharing ratio of YP samples was the highest. The YP population has shared haplotypes with all other populations except Brazil. Twenty-seven of the 51 YP haplotypes (52.9%) were shared with other populations, of which 59.3% (16/27), 44.4% (12/27), and 44.4% (12/27) were identical to some of the *Pvama-1* haplotypes observed in Thailand, Myanmar, and India populations, respectively. Haplotype 286 is the predominant haplotype in the YP population, with a frequency of 17.1%. Haplotype 17 is shared by most populations, consisting of YP, Myanmar, Thailand, Sri Lanka, Iran, and India populations. The haplotype network, drawn by excluding the 102 singletons from the analysis, showed that clusters from the Asian populations, Oceania, and South American overlapped (**Figure 5A**).

In addition, 77 unique haplotypes of CMB isolates were identified in 225 sequences (688 bp) from the 2009–2011, 2014–2016, and Myanmar populations. The PvAMA-1 gene information that was used for comparison in this study was from *P. vivax* isolates, which had been collected along the CMB area in Laiza, northeast Kachin State, Myanmar, in 2011–2012 (Li et al., 2013). Network analysis results of PvAMA-1 showed an obvious cluster for the three populations (**Figure S1A**). A total of 26.2% 2009–2011 haplotypes (11/42) were shared with the Myanmar (37) population, compared with 38.9% 2014–2016 haplotypes (7/18). Among them, the 2014–2016 population had the least haplotypes, which indicates less recombination in the CMB area.

Through the analysis by MEGA6, a total of 165 SNPs from the 10 populations (YP, Myanmar, Thailand, South Korea, Papua New Guinea, Sri Lanka, Iran, India, Venezuela, and Brazil) were detected. The results of principal component analysis (PCA) showed similar results with the network analysis, where only the Korea and PNG populations were partially separated from the global samples (**Figures 5B, C**).

Furthermore, the structure analysis results of global populations suggested 10 groups with optimized clusters value of *K* = 7 (**Figure 5D**). The results of structure analysis show that the YP and Thailand samples have the most *K* components (*n* = 7), followed by Myanmar and India (*n* = 6), Sri Lanka and Iran (*n* = 5), and PNG (*n* = 4) and the least *K* components (*n* = 3) in samples from Korea, Venezuela, and Brazil (**Figure 5D**). The distribution of *K* components in YP and Myanmar samples was significantly different (*χ*² = 70.207, *P* < 0.0001), although they all belong to the CMB area. Only the proportions of K2 component in YP and Myanmar samples are similar (21.71% and 19.17%, respectively) (*χ*² = 0.191, *P* = 0.66209). The *K* components of Myanmar samples were less than those in YP samples (**Figure 5D**), indicating that the YP population was more abundant than the Myanmar population. In addition, the migration rate between YP and Myanmar populations was estimated by Migrate-n version 4.4.3 (Beerli, 2006). The mean *Θ* values for YP and Myanmar populations were 0.06478 and 0.03100, respectively. The result showed asymmetric gene flow between YP and Myanmar populations, with the number of migrant individuals per generation (Nm) from Myanmar to YP (15.090) being higher than that from YP to Myanmar (11.189).

Meanwhile, the 2009–2011, 2014–2016, and Myanmar samples were analyzed by STRUCTURE software using no admixture model. A total of 62 SNPs from the three populations were detected by MEGA6. The structure results suggested three groups among the CMB samples with optimized clusters value of *K* = 7 (**Figure S1B**). The results of the structure analysis for the three populations show that the 2014–2016 samples generally lacked some *K* components that were commonly found in other samples. The distribution of K5 was particularly prominent as shown in **Figure S1B**. The distribution of K5 in 2009–2011 and 2014–2016 was significantly different (*χ*² = 15.439, *P* < 0.0001). Meanwhile, Myanmar samples also have fewer *K* components than those found in the 2009–2011 samples.

## Discussion

According to the statistical report of the WHO (2020), the malaria elimination target may not be achieved as expected by 2020. With the bottleneck of drug resistance to *P. vivax* (Imwong et al., 2007; Lu et al., 2010; Dayananda et al., 2018), new intervention tools and strategies are urgently needed to efficiently control and eliminate vivax malaria. For example, comprehensive research is needed to develop vaccine strategy. Though antigenic variation is one of the major challenges that affects the development of malaria vaccine (Esmaeili Rastaghi et al., 2014), advanced knowledge of parasite antigenic variants is a prerequisite for the rational design of a vaccine that might be efficient in various endemic areas.

In this study, we analyzed the genetic diversity of the PvAMA-1 gene from the border area of China and Myanmar and assessed its genetic variation across different time periods. Through the analysis of the full-length PvAMA-1 gene sequence, the results of Tajima’s *D* test and Fu and Li’s tests showed that there was no significant balancing selection in the 2009–2011 and 2014–2016 samples, which suggests that the PvAMA-1 gene was not under selection. However, the Ka/Ks values of PvAMA-1 were positive and statistically significant in all cases of the YP samples, which indicated that the PvAMA-1 of *P. vivax* populations in the CMB area was under significant positive selection during these times. Meanwhile, the linkage disequilibrium (*R* ^2^) results showed that there was more long-distance linkage in 2014–2016 than it was in 2009–2011. This indicates that the number of genetic recombinations in 2014–2016 was lower and more ancestor SNPs were retained, which is not consistent with the time periods. The 2014–2016 samples showed more long-distance linkage (**Figure 3**), which aligns with the growing scarcity of Chinese strains to participate in regional recombination. At the same time, the network analysis showed that the frequency of sharing haplotypes between 2014–2016 and Myanmar samples was higher than that in the 2009–2011 and Myanmar samples. This may indicate that the PvAMA-1 samples from the Chinese side of the China–Myanmar border were closer to the samples from the Myanmar side over time. Furthermore, the structure analysis showed that the 2014–2016 samples lacked some *K* components that were commonly found in other samples such as in the 2009–2011 samples (**Figure S1B**). This may indicate that the control of malaria transmission in China reduced the number and class of *P. vivax* spread in the CMB region, while the transmission from Myanmar continued. In addition, the movement of sections of the population that includes tourism, traveling and holidays, and border trade business, particularly in those free ports on the border, may also affect the population structure and genetic characteristics of malaria in this region (Cui et al., 2012). The structure analysis of PvAMA-1 in isolates from YP, Myanmar, Thailand, Korea, PNG, Sri Lanka, Iran, India, Venezuela, and Brazil revealed that the population structure in the CMB and Thailand is the most complex and abundant (**Figure 5D**). The study by Arnott et al. (2013) reported that diversity was the highest in PNG and Thailand, while it was the lowest in Venezuela. This suggests that there is greater genetic diversity of the PvAMA-1 gene in Southeast Asia, also including the CMB region. Although highly diverse, it was observed that the majority of the YP *Pvama-1* haplotypes (49%) are shared with Thailand, Myanmar, and India populations, with moderate *F*_ST_ values observed between YP, Myanmar, Thailand, and India isolates. This indicate that a YP-PvAMA-1-based multicomponent malaria vaccine may be effective in this entire region. Therefore, it is of great significance to explore the genetic diversity of PvAMA-1 in the CMB to provide instructive insights for the development of effective diagnostics and vaccines for *P. vivax*.

In a previous study, PvAMA-1 domains I and II covering the polymorphic regions have been shown to be major targets that elicit inhibitory responses (Remarque et al., 2008). In this study, Tajima’s *D* test showed that part of domain I (nt 401–500, Tajima’s *D*: 2.233, *P* < 0.05) of PvAMA-1 was under positive balancing selection in the YP population. Similar findings have been reported from other studies where highly significant positive values have been consistently observed within domain I of the Myanmar, PNG, Iran, India, and Venezuela populations (Ord et al., 2008; Arnott et al., 2013; Zakeri et al., 2013; Zhu et al., 2016; Kale et al., 2021). Such informative findings suggest this domain a dominant target of host immune responses. Domains II and III showed direction selection in YP samples through some time periods different from those of Myanmar samples, in which all PvAMA-1 domains were balancing selected (Zhu et al., 2016). The domain II of *Pvama-1* has been reported as highly immunogenic (Pizarro et al., 2005; Múfalo et al., 2008; Remarque et al., 2008; Gentil et al., 2010). In addition, positive selection within domain II of *Pvama-1* populations from Sri Lanka has been observed (Gunasekera et al., 2007). However, in this study, no evidence was found for diversifying selection on domains II and III of *Pvama-1* from the CMB area as confirmed by neutrality tests, which is in agreement with previous reports (Ord et al., 2008; Arnott et al., 2013).

Sequences of *Pvama-1* from the YP population were compared to the reference sequence (PVX_092275), and 61 SNPs resulting in 40 amino acid substitutions were identified in the YP *Pvama-1*. Most mutation loci were found in domain I of PvAMA-1 from YP samples, which aligns with the findings in other populations as previously reported (Gunasekera et al., 2007; Ord et al., 2008; Putaporntip et al., 2009; Arnott et al., 2013; Zakeri et al., 2013; Kang et al., 2015; Zhu et al., 2016; Bittencourt et al., 2020; Kale et al., 2021). Meanwhile, the AMA-1 ligand-binding site and a major target of protective immunity have been proven to be a hydrophobic trough composed of domains I and II (Coley et al., 2007). The polymorphic residues 197, 200, 201, 204, and 225 have been proven to be important for PvAMA-1 binding (Coley et al., 2007). However, no mutations were found in these important amino acid loci in the YP samples used in this study.

In this study, the mutation frequencies of 10 mutation sites were more than 50% (**Table 2**). Most importantly, the six tightly preserved amino acid changes (D107, R112, K120, E145, E277, and R438), which are the most outstanding characteristics found in the YP PvAMA-1 analyzed in this study, were well-preserved in all the populations except that from Korea, which revealed some inconsistent amino acid sites (Kang et al., 2015; Bittencourt et al., 2020). Meanwhile, when compared with other populations, PvAMA-1 of YP had more high-frequency mutation sites. These results also suggest that PvAMA-1 from the CMB area showed different patterns of polymorphic nature compared with those from other geographical areas, and more abundant genetic diversity was observed among isolates globally (Arnott et al., 2013; Kang et al., 2015; Zhu et al., 2016).

Some of the SNPs identified in *P. vivax* isolates globally, including E145K, P210S, R249H, G253E, K352E, R438H, and N445D, overlap with the B-cell epitope regions. These amino acid changes may affect the protein structure by causing changes in charge and polarity of the protein and might help parasites to escape from host immunity (Anders et al., 1998). In this study, except the R249H, other mutations were all observed (**Table 2**) in PvAMA-1 which contained more immune escape-related mutations than those of PvAMA-1 from other reported populations (Kang et al., 2015). This indicates that *P. vivax* in the CMB area is under strong immune pressure.

Collectively, we found a high diversity and a complex population structure of PvAMA-1 in the CMB region of Yunnan Province, China, in comparison with the PvAMA-1 from other populations. The study revealed the unique genetic diversity of *Pvama-1* in the CMB area, which is an instructive finding for the development of extensive and effective malaria vaccines.

## Conclusion

This study provides the first in-depth understanding of the genetic diversity of PvAMA-1 from different time periods in the CMB. PvAMA-1 domain I is the dominant target of positive diversifying selection. Meanwhile, the majority of the YP *Pvama-1* haplotypes, shared with Thailand, Myanmar, and India populations, indicate the possibility of a YP-PvAMA-1-based multicomponent malaria vaccine with an effect on this entire region. These results suggest PvAMA-1 a dominant target of host immune selection and a potential vaccine target.

## Data Availability Statement

All materials and data supporting these findings are contained within the manuscript and supplementary figure and table. The sequences have been deposited in the GenBank database under the accession numbers OK605600 - OK605751 for the China-Myanmar border isolates in Yunnan Province of China.

## Ethics Statement

This study was conducted according to the principles expressed in the Declaration of Helsinki. Before blood collection, the study protocol, potential risks and benefits were explained to the participants, and written informed consent from all adult participants and from the parents, or legal guardians of children. Blood was collected following institutional ethical guidelines reviewed and approved by the ethics committee at National Institute of Parasitic Diseases, Chinese Center for Disease Control and Prevention (no. 20120826).

## Author Contributions

Conceived and designed the experiments: Y-BC, J-HC, J-HW, and YW. Performed the experiments: Y-BC, S-BC, T-QS, BX, and YW. Analyzed the data: Y-BC, H-MS, KK, and YW. Contributed the reagents/materials/analysis tools: S-BC, KK, T-QS, BX, and YW. Wrote the paper: Y-BC, J-HC, J-HW, and YW. All authors contributed to the article and approved the submitted version.

## Funding

This work was financially supported in part by the Fifth Round of Three-Year Public Health Action Plan of Shanghai (Grant No. GWV-10.1-XK13), the Opening Project of Key Laboratory of Ministry of Education for Environmental Pollution and Disease Monitoring (Grant No. GMU-2017-HJZ-02), the National Sharing Service Platform for Parasite Resources (Grant No. TDRC-2019-194-30), the Shanghai Municipal Health Commission Planning (Grant No. 201840007), the Zhejiang Provincial Natural Science Foundation of China (Grant No. LY17H190005), and the Foundation of National Science and Technology Major Program (Grant no. 2012ZX10004-220). The funding bodies had no role in the design of the study; the collection, analysis, and interpretation of data; or in the writing of the manuscript.

## Conflict of Interest

The authors declare that the research was conducted in the absence of any commercial or financial relationships that could be construed as a potential conflict of interest.

## Publisher’s Note

All claims expressed in this article are solely those of the authors and do not necessarily represent those of their affiliated organizations, or those of the publisher, the editors and the reviewers. Any product that may be evaluated in this article, or claim that may be made by its manufacturer, is not guaranteed or endorsed by the publisher.

## Supplementary Material

Supplementary Table 1 The distribution of amino acid mutation sites (R66-N445) in eleven populations.

Supplementary Figure 1 Network and structure analyses of PvAMA-1 in isolates from CMB. **(A)** The proportion of Pv*ama-1* haplotype variations observed in different populations. Samples are coloured according to different populations. **(B)** Structure analysis of the full set of variation loci from all isolates. Cluster for each isolate was assessed according to an optimized cluster value of *K* = 7.

## References

[B1] Anders R. F. Crewther P. E. Edwards S. Margetts M. Matthew M. L. Pollock B.. (1998). Immunisation With Recombinant AMA-1 Protects Mice Against Infection With Plasmodium Chabaudi. Vaccine 16, 240–247. doi: 10.1016/s0264-410x(97)88331-4 9607037

[B2] Arnott A. Mueller I. Ramsland P. A. Siba P. M. Reeder J. C. Barry A. E. (2013). Global Population Structure of the Genes Encoding the Malaria Vaccine Candidate, Plasmodium Vivax Apical Membrane Antigen 1 (Pvama1). PloS Negl. Trop. Dis. 7, e2506. doi: 10.1371/journal.pntd.0002506 24205419 PMC3814406

[B3] Balloux F. Lugon-Moulin N. (2002). The Estimation of Population Differentiation With Microsatellite Markers. Mol. Ecol. 11, 155–165. doi: 10.1046/j.0962-1083.2001.01436.x 11856418

[B4] Bandelt H. J. Forster P. Röhl A. (1999). Median-Joining Networks for Inferring Intraspecific Phylogenies. Mol. Biol. Evol. 16, 37–48. doi: 10.1093/oxfordjournals.molbev.a026036 10331250

[B5] Bargieri D. Y. Andenmatten N. Lagal V. Thiberge S. Whitelaw J. A. Tardieux I.. (2013). Apical Membrane Antigen 1 Mediates Apicomplexan Parasite Attachment But Is Dispensable for Host Cell Invasion. Nat. Commun. 4, 2552. doi: 10.1038/ncomms3552 24108241 PMC3826631

[B6] Beerli P. (2006). Comparison of Bayesian and Maximum-Likelihood Inference of Population Genetic Parameters. Bioinformatics 22 (3), 341–345. doi: 10.1093/bioinformatics/bti803 16317072

[B7] Beeson J. G. Kurtovic L. Dobaño C. Opi D. H. Chan J. A. Feng G.. (2019). Challenges and Strategies for Developing Efficacious and Long-Lasting Malaria Vaccines. Sci. Transl. Med. 11 (474), eaau1458. doi: 10.1126/scitranslmed.aau1458 30626712

[B8] Bittencourt N. C. Silva A.B. I. E. D. Virgili N. S. Schappo A. P. Gervásio J. H. D. B. Pimenta T. S.. (2020). *Plasmodium Vivax* AMA1: Implications of Distinct Haplotypes for Immune Response. PloS Negl. Trop. Dis. 14 (7), e0008471. doi: 10.1371/journal.pntd.0008471 32639964 PMC7371208

[B9] Brashear A. M. Fan Q. Hu Y. Li Y. Zhao Y. Wang Z.. (2020). Population Genomics Identifies a Distinct Plasmodium Vivax Population on the China Myanmar Border of Southeast Asia. PloS Negl. Trop. Dis. 14 (8), e0008506. doi: 10.1371/journal.pntd.0008506 32745103 PMC7425983

[B10] Chen S. B. Wang Y. Kassegne K. Xu B. Shen H. M. Chen J. H. (2017). Whole-Genome Sequencing of a Plasmodium Vivax Clinical Isolate Exhibits Geographical Characteristics and High Genetic Variation in China-Myanmar Border Area. BMC Genomics 18 (1), 131. doi: 10.1186/s12864-017-3523-y 28166727 PMC5294834

[B11] Coley A. M. Gupta A. Murphy V. J. Bai T. Kim H. Foley M.. (2007). Structure of the Malaria Antigen AMA1 in Complex With a Growth-Inhibitory Antibody. PloS Pathog. 3 (9), 1308–1319. doi: 10.1371/journal.ppat.0030138 17907804 PMC2323298

[B12] Cui L. W. Yan G. Y. Sattabongkot J. Chen B. Cao Y. M. Fan Q.. (2012). Challenges and Prospects for Malaria Elimination in the Greater Mekong Subregion. Acta Trop. 121 (3), 240–245. doi: 10.1016/j.actatropica.2011.04.006 21515238 PMC3155744

[B13] Dayananda K. K. Achur R. N. Gowda D. C. (2018). Epidemiology, Drug Resistance, and Pathophysiology of Plasmodium Vivax Malaria. J. Vector Borne Dis. 55 (1), 1–8. doi: 10.4103/0972-9062.234620 29916441 PMC6996296

[B14] Esmaeili Rastaghi A. R. Nedaei F. Nahrevanian H. Hoseinkhan N. (2014). Genetic Diversity and Effect of Selection at Apical Membrane Antigen-1 (AMA-1) Among Iranian *Plasmodium Vivax* Isolates. Folia Parasitol (Praha) 61 (5), 385–393. doi: 10.14411/fp.2014.048 25549495

[B15] Evanno G. Regnaut S. Goudet J. (2005). Detecting the Number of Clusters of Individuals Using the Software STRUCTURE: A Simulation Study. Mol. Ecol. 14 (8), 2611–2620. doi: 10.1111/j.1365-294X.2005.02553.x 15969739

[B16] Excoffier L. Lischer H. E. (2010). Arlequin Suite Ver 3.5: A New Series of Programs to Perform Population Genetics Analyses Under Linux and Windows. Mol. Ecol. Resour. 10, 564–567. doi: 10.1111/j.1755-0998.2010.02847.x 21565059

[B17] Feng J. Xiao H. Zhang L. Yan H. Feng X. Fang W.. (2015). The Plasmodium Vivax in China: Decreased in Local Cases But Increased Imported Cases From Southeast Asia and Africa. Sci. Rep. 5, 8847. doi: 10.1038/srep08847 25739365 PMC4350086

[B18] Flannery E. L. Markus M. B. Vaughan A. M. (2019). Plasmodium Vivax. Trends Parasitol. 35 (7), 583–584. doi: 10.1016/j.pt.2019.04.005 31176582

[B19] Gaur D. Mayer D. C. G. Miller L. H. (2004). Parasite Ligand-Host Receptor Interactions During Invasion of Erythrocytes by Plasmodium Merozoites. Int. J. Parasitol. 34, 1413–1429. doi: 10.1016/j.ijpara.2004.10.010 15582519

[B20] Gentil F. Bargieri D. Y. Leite J. A. Françoso K. S. Patricio M. B. M. Espíndola N. M.. (2010). A Recombinant Vaccine Based on Domain II of Plasmodium Vivax Apical Membrane Antigen 1 Induces High Antibody Titres in Mice. Vaccine 28, 6183–6190. doi: 10.1016/j.vaccine.2010.07.017 20654667

[B21] Gunasekera A. M. Wickramarachchi T. Neafsey D. E. Ganguli I. Perera L. Premaratne P. H.. (2007). Genetic Diversity and Selection at the Plasmodium Vivax Apical Membrane Antigen-1 (PvAMA-1) Locus in a Sri Lankan Population. Mol. Biol. Evol. 24 (4), 939–947. doi: 10.1093/molbev/msm013 17244598

[B22] Howes R. E. Battle K. E. Mendis K. N. Smith D. L. Cibulskis R. E. Baird J. K.. (2016). Global Epidemiology of Plasmodium Vivax. Am. J. Trop. Med. Hyg. 95 (6 Suppl), 15–34. doi: 10.4269/ajtmh.16-0141 PMC5198891 27402513

[B23] Imwong M. Snounou G. Pukrittayakamee S. Tanomsing N. Kim J. R. Nandy A.. (2007). Relapses of Plasmodium Vivax Infection Usually Result From Activation of Heterologous Hypnozoites. J. Infect. Dis. 195 (7), 927–933. doi: 10.1086/512241 17330781

[B24] Joshi H. (2003). Markers for Population Genetic Analysis of Human Plasmodia Species, P. Falciparum and *P. Vivax* . J. Vector Borne Dis. 40 (3-4), 78–83. doi: 10.1186/cc5137 15119076

[B25] Kale S. Pande V. Singh O. P. Carlton J. M. Mallick P. K. (2021). Genetic Diversity in Two Leading Plasmodium Vivax Malaria Vaccine Candidates AMA1 and MSP1_19_ at Three Sites in India. PloS Negl. Trop. Dis. 15 (8), e0009652. doi: 10.1371/journal.pntd.0009652 34370745 PMC8376102

[B26] Kang J. M. Lee J. Cho P. Y. Moon S. U. Ju H. L. Ahn S. K.. (2015). Population Genetic Structure and Selection of Apical Membrane Antigen-1 in Plasmodium Vivax Korean Isolates. Malar J. 14, 455. doi: 10.1186/s12936-015-0942-6 26572984 PMC4647566

[B27] Kassegne K. Komi Koukoura K. Shen H. M. Chen S. B. Fu H. T. Chen Y. Q.. (2020). Genome-Wide Analysis of the Malaria Parasite Plasmodium Falciparum Isolates From Togo Reveals Selective Signals in Immune Selection-Related Antigen Genes. Front. Immunol. 11, 552698. doi: 10.3389/fimmu.2020.552698 33193320 PMC7645038

[B28] Kusi K. A. Faber B. W. Thomas A. W. Remarque E. J. (2009). Humoral Immune Response to Mixed PfAMA1 Alleles; Multivalent PfAMA1 Vaccines Induce Broad Specificity. PloS One 4 (12), e8110. doi: 10.1371/journal.pone.0008110 19956619 PMC2779588

[B29] Lai S. J. Sun J. L. Ruktanonchai N. W. Zhou S. Yu J. X. Routledge I.. (2019). Changing Epidemiology and Challenges of Malaria in China Towards Elimination. Malar J. 18 (1), 107. doi: 10.1186/s12936-019-2736-8 30922301 PMC6440015

[B30] Lamarque M. Besteiro S. Papoin J. Roques M. Vulliez-Le Normand B. Morlon-Guyot J.. (2011). The RON2-AMA1 Interaction Is a Critical Step in Moving Junction-Dependent Invasion by Apicomplexan Parasites. PloS Pathog. 7 (2), e1001276. doi: 10.1371/journal.ppat.1001276 21347343 PMC3037350

[B31] Librado P. Rozas J. (2009). DnaSP V5: A Software for Comprehensive Analysis of DNA Polymorphism Data. Bioinformatics 25 (11), 1451–1452. doi: 10.1093/bioinformatics/btp187 19346325

[B32] Li N. Parker D. M. Yang Z. Q. Fan Q. Zhou G. F. Ai G. P.. (2013). Risk Factors Associated With Slide Positivity Among Febrile Patients in a Conflict Zone of Northeastern Myanmar Along the China-Myanmar Border. Malar J. 12, 361. doi: 10.1186/1475-2875-12-361 24112638 PMC3852943

[B33] Lu F. Lim C. S. Nam D. H. Kim K. Lin K. Kim T. S.. (2010). Mutations in the Antifolate-Resistance-Associated Genes Dihydrofolate Reductase and Dihydropteroate Synthase in Plasmodium Vivax Isolates From Malaria-Endemic Countries. Am. J. Trop. Med. Hyg. 83 (3), 474–479. doi: 10.4269/ajtmh.2010.10-0004 20810806 PMC2929037

[B34] Martin D. P. Murrell B. Golden M. Khoosal A. Muhire B. (2015). RDP4: Detection and Analysis of Recombination Patterns in Virus Genomes. Virus Evol. 1, vev003. doi: 10.1093/ve/vev003 27774277 PMC5014473

[B35] Maynard Smith J. (1992). Analyzing the Mosaic Structure of Genes. J. Mol. Evol. 34 (2), 126–129. doi: 10.1007/BF00182389 1556748

[B36] Mueller I. Adams J. H. (2017). The Biology of Plasmodium Vivax. Cold Spring Harb. Perspect. Med. 7 (9), a025585. doi: 10.1101/cshperspect.a025585 28490540 PMC5580510

[B37] Mueller I. Galinski M. R. Baird J. K. Carlton J. M. Kochar D. K. Alonso P. L.. (2009). Key Gaps in the Knowledge of Plasmodium Vivax, a Neglected Human Malaria Parasite. Lancet Infect. Dis. 9 (9), 555–566. doi: 10.1016/S1473-3099(09)70177-X 19695492

[B38] Mueller I. Shakri A. R. Chitnis C. E. (2015). Development of Vaccines for Plasmodium Vivax Malaria. Vaccine 33 (52), 7489–7495. doi: 10.1016/j.vaccine.2015.09.060 26428453

[B39] Múfalo B. C. Gentil F. Bargieri D. Y. Costa F. T. M. Rodrigues M. M. Soares I. S. (2008). Plasmodium Vivax Apical Membrane Antigen-1: Comparative Recognition of Different Domains by Antibodies Induced During Natural Human Infection. Microbes Infect. 10 (12-13), 1266–1273. doi: 10.1016/j.micinf.2008.07.023 18692152

[B40] Murhandarwati E. E. H. Herningtyas E. H. Puspawati P. Mau F. Chen S. B. Shen H. M.. (2020). Genetic Diversity of Merozoite Surface Protein 1–42 (MSP1-42) Fragment of Plasmodium Vivax From Indonesian Isolates: Rationale Implementation of Candidate MSP1 Vaccine. Infect. Genet. Evol. 85, 104573. doi: 10.1016/j.meegid.2020.104573 32987191

[B41] Nei M. Gojobori T. (1986). Simple Methods for Estimating the Numbers of Synonymous and Nonsynonymous Nucleotide Substitutions. Mol. Biol. Evol. 3 (5), 418–426. doi: 10.1093/oxfordjournals.molbev.a040410 3444411

[B42] Ord R. L. Tami A. Sutherland C. J. (2008). *Ama1* Genes of Sympatric Plasmodium Vivax and *P. Falciparum* From Venezuela Differ Significantly in Genetic Diversity and Recombination Frequency. PloS One 3 (10), e3366. doi: 10.1371/journal.pone.0003366 18846221 PMC2559863

[B43] Pizarro J. C. Vulliez-Le Normand B. Chesne-Seck M. L. Collins C. R. Withers-Martinez C. Hackett F.. (2005). Crystal Structure of the Malaria Vaccine Candidate Apical Membrane Antigen 1. Science 308 (5720), 408–411. doi: 10.1126/science.1107449 15731407

[B44] Putaporntip C. Jongwutiwes S. Grynberg P. Cui L. W. Hughes A. L. (2009). Nucleotide Sequence Polymorphism at the Apical Membrane Antigen-1 Locus Reveals Population History of Plasmodium Vivax in Thailand. Infect. Genet. Evol. 9 (6), 1295–1300. doi: 10.1016/j.meegid.2009.07.005 19643205 PMC2790030

[B45] Remarque E. J. Faber B. W. Kocken C. H. Thomas A. W. (2008). Apical Membrane Antigen 1: A Malaria Vaccine Candidate in Review. Trends Parasitol. 24 (2), 74–84. doi: 10.1016/j.pt.2007.12.002 18226584

[B46] Richard D. MacRaild C. A. Riglar D. T. Chan J. A. Foley M. Baum J.. (2010). Interaction Between Plasmodium Falciparum Apical Membrane Antigen 1 and the Rhoptry Neck Protein Complex Defines a Key Step in the Erythrocyte Invasion Process of Malaria Parasites. J. Biol. Chem. 285 (19), 14815–14822. doi: 10.1074/jbc.M109.080770 20228060 PMC2863225

[B47] Srinivasan P. Beatty W. L. Diouf A. Herrera R. Ambroggio X. Moch J. K.. (2011). Binding of Plasmodium Merozoite Proteins RON2 and AMA1 Triggers Commitment to Invasion. Proc. Natl. Acad. Sci. U.S.A. 108 (32), 13275–13280. doi: 10.1073/pnas.1110303108 21788485 PMC3156155

[B48] Tamura K. Stecher G. Peterson D. Filipski A. Kumar S. (2013). MEGA6: Molecular Evolutionary Genetics Analysis Version 6.0. Mol. Biol. Evol. 30 (12), 2725–2729. doi: 10.1093/molbev/mst197 24132122 PMC3840312

[B49] Triglia T. Healer J. Caruana S. R. Hodder A. N. Anders R. F. Crabb B. S.. (2000). Apical Membrane Antigen 1 Plays a Central Role in Erythrocyte Invasion by Plasmodium Species. Mol. Microbiol. 38 (4), 706–718. doi: 10.1046/j.1365-2958.2000.02175.x 11115107

[B50] Vogel G. (2013). The Forgotten Malaria. Science 342 (6159), 684–687. doi: 10.1126/science.342.6159.684 24202156

[B51] von Seidlein L. Peerawaranun P. Mukaka M. Nosten F. H. Nguyen T. N. Hien T. T.. (2019). The Probability of a Sequential Plasmodium Vivax Infection Following Asymptomatic *Plasmodium Falciparum* and *P. Vivax* Infections in Myanmar, Vietnam, Cambodia, and Laos. Malar J. 18 (1), 449. doi: 10.1186/s12936-019-3087-1 31888643 PMC6937799

[B52] Wang D. P. Zhang Y. B. Zhang Z. Zhu J. Yu J. (2010). KaKs_Calculator 2.0: A Toolkit Incorporating Gamma-Series Methods and Sliding Window Strategies. Genomics Proteomics Bioinf. 8 (1), 77–80. doi: 10.1016/S1672-0229(10)60008-3 PMC5054116 20451164

[B53] White N. J. Imwong M. (2012). Relapse. Adv. Parasitol. 80, 113–150. doi: 10.1016/B978-0-12-397900-1.00002-5 23199487

[B54] WHO (2020). World Malaria Report 2020. Geneva: World Health Organization.

[B55] Yang Z. Nielsen R. (2000). Estimating Synonymous and Nonsynonymous Substitution Rates Under Realistic Evolutionary Models. Mol. Biol. Evol. 17 (1), 32–43. doi: 10.1093/oxfordjournals.molbev.a026236 10666704

[B56] Zakeri S. Sadeghi H. Mehrizi A. A. Djadid N. D. (2013). Population Genetic Structure and Polymorphism Analysis of Gene Encoding Apical Membrane Antigen-1 (AMA-1) of Iranian Plasmodium Vivax Wild Isolates. Acta Trop. 126 (3), 269–279. doi: 10.1016/j.actatropica.2013.02.017 23467011

[B57] Zhang S. S. Zhou S. S. Zhou Z. B. Chen T. M. Wang X. Z. Shi W. Q.. (2018). Monitoring of Malaria Vectors at the China-Myanmar Border While Approaching Malaria Elimination. Parasit. Vectors 11 (1), 511. doi: 10.1186/s13071-018-3073-4 30219093 PMC6139178

[B58] Zhou X. Huang J. L. Njuabe M. T. Li S. G. Chen J. H. Zhou X. N. (2014). A Molecular Survey of Febrile Cases in Malaria-Endemic Areas Along China-Myanmar Border in Yunnan Province, People’s Republic of China. Parasite 21, 27. doi: 10.1051/parasite/2014030 24954235 PMC4066189

[B59] Zhu X. T. Zhao P. Wang S. Liu F. Liu J. Wang J.. (2016). Analysis of Pvama1 Genes From China-Myanmar Border Reveals Little Regional Genetic Differentiation of *Plasmodium Vivax* Populations. Parasit. Vectors 9 (1), 614. doi: 10.1186/s13071-016-1899-1 27899135 PMC5129220

