# Supplementary material for: Genetic Diversity and Selection of Plasmodium vivax Apical Membrane Antigen-1 in China–Myanmar Border of Yunnan Province, China, 2009–2016
Source: Front Cell Infect Microbiol. 2022 Jan 5;11:742189. doi: 10.3389/fcimb.2021.742189 (PMC8766981; doi:10.3389/fcimb.2021.742189)
Supplement: Supplementary Table 1 — The distribution of amino acid mutation sites (R66-N445) in eleven populations. [file Table_1.doc]

**Table S1. The distribution of amino acid mutation sites (R66-N445) in eleven populations**

|  | 2009-2011 | 2014-2016 | Myanmar | Iran | Korea | PNG | Sri Lanka | Thailand | Venezuela | India | Brazil | Position |
| --- | --- | --- | --- | --- | --- | --- | --- | --- | --- | --- | --- | --- |
| R66K | + | - | - | + | - | + | - | - | + | + | + |  |
| V102D | + | - | - | - | - | - | - | - | - | - | - | * |
| D107N/A | + | + | + | + | + | + | + | + | + | + | + | * |
| R112K/T | + | + | + | + | + | + | + | + | + | + | + | * |
| G117R | + | - | + | - | - | - | - | + | - | - | - | * |
| K120R | + | + | + | + | + | + | + | + | + | + | + | * |
| N130K | + | - | + | + | - | + | - | + | + | + | + | * |
| N132D | + | + | + | + | + | + | + | + | + | + | + | * |
| L140I | + | + | + | + | - | + | + | + | + | + | + | * |
| A141E | + | + | + | + | + | + | + | + | + | + | + | * |
| E145A | + | + | + | + | + | + | + | + | + | + | + | * |
| K188E | + | - | - | + | - | - | + | - | - | + | - | * |
| E189K/N | + | + | + | + | + | + | + | + | + | + | + | * |
| K190E | + | + | + | + | - | + | + | + | + | + | + | * |
| T191K | + | + | - | - | + | - | - | - | - | - | - | * |
| H193Y | + | - | - | + | - | - | + | + | + | + | - | * |
| P210S | + | + | + | + | - | + | + | + | + | + | - | * |
| V218L | + | - | + | - | - | - | + | + | - | + | - | * |
| E227K/V | + | + | + | + | + | + | + | + | - | + | + | * |
| S228N/D | + | + | + | + | + | + | + | + | - | + | + | * |
| G253E | + | + | + | + | - | + | + | + | - | + | - |  |
| K256Q | + | - | - | - | - | - | - | - | - | - | - |  |
| E277K | + | + | + | + | + | + | + | + | + | + | + | ** |
| G288E | + | + | + | + | - | + | + | + | + | + | - | ** |
| P295S | + | - | - | - | - | - | - | - | - | - | - | ** |
| N316T | - | + | - | - | - | - | - | - | - | - | - | ** |
| M319I | - | + | + | - | - | - | - | - | - | - | - | ** |
| K336R | - | + | - | - | - | - | - | - | - | - | - | ** |
| K352N/E | + | + | + | + | - | + | + | + | + | + | + | ** |
| K368I | + | + | - | + | - | - | + | + | + | + | + |  |
| Q380K/R | + | + | + | + | - | + | + | + | + | + | + |  |
| V382E | + | + | + | + | - | - | - | + | - | + | + |  |
| L384P/R | + | + | + | + | + | + | + | + | + | + | + |  |
| E385D/Q/K | + | + | + | + | + | + | + | + | - | + | - |  |
| K400R | + | + | - | + | - | + | + | + | - | + | - | *** |
| R438H | + | + | + | + | - | + | + | + | + | + | + | *** |
| N445D | + | + | + | + | + | - | - | - | + | - | + | *** |
| n (37) | 34 | 28 | 26 | 28 | 14 | 23 | 25 | 27 | 21 | 28 | 21 |  |

“-” no mutation, “+” mutation, “*” Domain I, “**” Domain II, “***” Domain III. Reference sequence, “n” number of mutations, PVX_092275.
