# Supplementary figures and images for: Genetic Diversity and Selection of Plasmodium vivax Apical Membrane Antigen-1 in China–Myanmar Border of Yunnan Province, China, 2009–2016
Source: Front Cell Infect Microbiol. 2022 Jan 5;11:742189. doi: 10.3389/fcimb.2021.742189 (PMC8766981; doi:10.3389/fcimb.2021.742189)

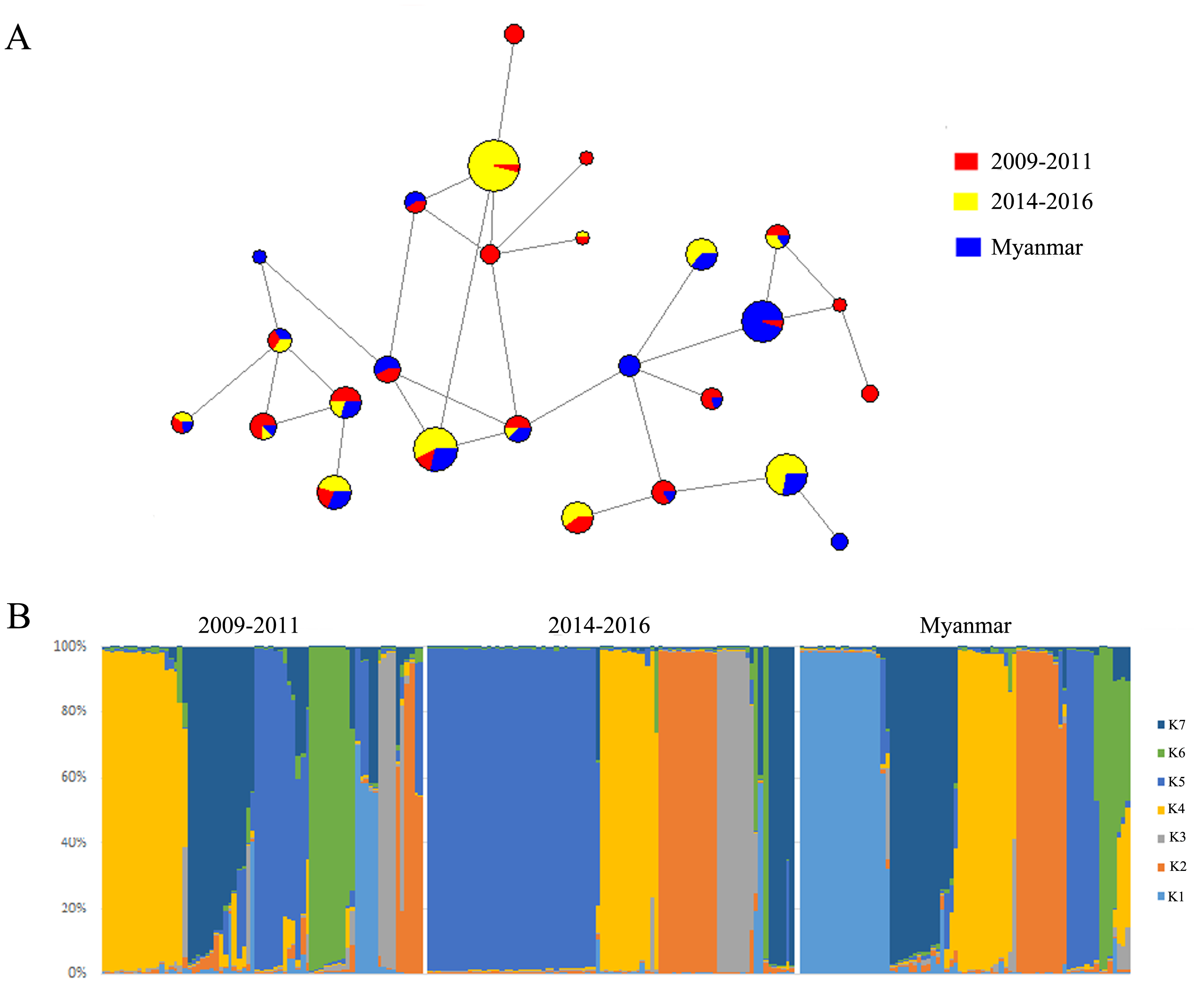

Supplement: Supplementary Figure 1 — Network and structure analyses of PvAMA-1 in isolates from CMB. (A) The proportion of Pvama-1 haplotype variations observed in different populations. Samples are coloured according to different populations. (B) Structure analysis of the full set of variation loci from all isolates. Cluster for each isolate was assessed according to an optimized cluster value of K = 7. [file Image_1.tif]
